# Encapsulated Fecal Microbiota Transplantation: Development, Efficacy, and Clinical Application

**DOI:** 10.3389/fcimb.2022.826114

**Published:** 2022-03-17

**Authors:** Hossam F. Halaweish, Sonja Boatman, Christopher Staley

**Affiliations:** ^1^ Division of Basic & Translational Research, Department of Surgery, University of Minnesota, Minneapolis, MN, United States; ^2^ BioTechnology Institute, University of Minnesota, Saint Paul, MN, United States

**Keywords:** gut microbiota, fecal microbiota transplant (FMT), microbial ecology, pharmacology, pharmacokinetics

## Abstract

Fecal microbiota transplantation (FMT) has been established as a highly restorative therapeutic approach for treating recurrent *Clostridioides difficile* infection (rCDI). Recently, the use of capsule-based fecal microbiota transplantation (cFMT) has been shown to be a clinically effective approach to restore intestinal microbiota composition. This convenient, oral delivery provides an easy route of administration and a newfound flexibility for clinicians and patients. In this review, we discuss the development of cFMT, paying particular attention to lyophilized cFMT products. We review the available published clinical studies comparing cFMT with lower endoscopic FMT (eFMT) or placebo. We further discuss the pharmacokinetics of FMT, which should be understood in a framework of microbial ecology that considers the complex and dynamic interactions of gut microbiota with host factors and other microorganisms. Promisingly, the results of multiple trials investigating cFMT *vs*. eFMT in rCDI show cFMT to be as effective as eFMT at preventing rCDI. However, its efficacy in non-rCDI conditions, including obesity and metabolic syndrome, inflammatory bowel disease, HIV, and neurologic conditions, is less clear and more research is needed in these areas. Standardization of formulation, dose, and timing of administration to ensure optimal microbiota engraftment and clinical response is also a challenge to be addressed. Overall, cFMT is a practical method for fecal microbiota transplantation, with similar efficacy to eFMT in the resolution of rCDI, that holds therapeutic potential in a variety of other diseases.

## Background

The intestinal (gut) microbiome is defined as the complete collection of microorganisms, including bacteria, viruses, protozoa, and fungi, in addition to their collective genetic material that is present in the gastrointestinal tract (Shreiner et al., 2015). The trillions of gut bacterial cells can be further classified into thousands of different species including more than 5,000 bacterial strains. The gut microbiota is crucial in maintaining a healthy gut; this biological system produces the essential vitamins B12 and K and digests and metabolizes nutrients from ingested substances such as complex polysaccharides (e.g., fiber) and medications (Shreiner et al., 2015). Importantly, the commensal microbiota also provides protection from pathogen colonization through Toll-like receptor-mediated immune activation (Brandl et al., 2007), modulation of host metabolites (Nagao-Kitamoto et al., 2020), and production of bactericidal compounds (Coyne et al., 2019). Indirectly, the competitive exclusion of pathogens *via* niche specialization and efficient consumption of available nutrients also occurs. Given these and other roles extending beyond the gastrointestinal tract, the microbiota is now recognized as a critical component of health and disease (Lozupone et al., 2012; Marchesi et al., 2016).

Factors such as environment, diet, host genetics, and medications contribute to a person’s unique microbial composition (Lozupone et al., 2012; Lloyd-Price et al., 2017). Medications, especially antibiotics, can have an adverse effect on the gut microbiome, and the extensive use of antibiotics in medicine over the last few decades has contributed to the depletion of the gut microbiota and led to the increased development of antibiotic-resistant pathogens (Fischbach and Walsh, 2009; Modi et al., 2014). Furthermore, the rapid, diminishing diversity of the gut microbiota following antibiotic exposure directly causes a loss of function and structure of the microbial community (Dethlefsen et al., 2008; Dethlefsen and Relman, 2011). Both antibiotic-resistant pathogens as well as the decreased diversity are gaining recognition as prominent health concerns related to the use and overuse of antibiotics (Khoruts and Sadowsky, 2016).

### Fecal Microbiota Transplantation

In response to issues associated with antibiotic use described above, fecal microbiota transplantation (FMT) has reemerged as a restorative therapeutic approach and is especially recognized for treating recurrent *Clostridioides difficile* infection (rCDI) once antibiotics have proven ineffective (Hamilton et al., 2012; Borody et al., 2013; van Nood et al., 2013). This procedure involves the transplantation of the gut microbiota from a healthy donor to a patient to restore normal diversity and function. FMT results in donor-like normalization of the gut microbial community structure and functionality without causing dysbiosis associated with antibiotic treatments, a predominant causal risk factor for rCDI in most patients (Weingarden et al., 2015; Hui et al., 2019). Use of FMT has shown a high rate of efficacy (~90%) when treating rCDI (Drekonja et al., 2015).

FMT has evolved over the last decade toward the use of increasingly regulated and standardized products that are more easily integrated into mainstream clinical practice (Hamilton et al., 2012; Khoruts et al., 2021). Traditionally, the United States has performed FMT *via* a lower endoscopic route of administration (eFMT), which has the advantage of direct application to the colon (Hamilton et al., 2012). In Europe, administration through a nasogastric or nasoduodenal tube (NGT/NDT) is more commonly done (van Nood et al., 2013). In a study that aimed at comparing the clinical efficacy of both routes, it was found that the rCDI cure rates did not significantly differ between methods (Postigo and Kim, 2012; Youngster et al., 2014a), although more recent accounts suggest the superiority of colonoscopic delivery (Ramai et al., 2020). Despite the procedural differences present in the routes of administration, FMT *via* NGT or colonoscopy appears to be safe and highly effective for the resolution and management of CDI (Postigo and Kim, 2012; Ramai et al., 2020).

### Clinical Efficacy of Capsule-Based FMT

The push toward clinical practicality and flexibility has recently resulted in the development of an oral FMT administration route using encapsulated frozen or freeze-dried material (cFMT) (Youngster et al., 2014a; Staley et al., 2017a). This oral preparation is preferable for many patients and providers due to its greater ease of administration and less invasive nature (Kao et al., 2017). The efficacy of cFMT is an area of ongoing study, and in the setting of rCDI, multiple recent trials (Youngster et al., 2014b; Kao et al., 2017; Jiang et al., 2018; Ramai et al., 2020) have demonstrated comparable clinical results of cFMT to eFMT (**Table 1**). Results from these trials showed the non-inferiority of cFMT compared to eFMT with 84%-96% clinical resolution of rCDI in patients treated with capsules and no significant difference in the rate of adverse events. In a recent meta-analysis of 26 studies, including 16 that administered FMT *via* colonoscopy and four that used capsules, both routes of administration achieved equivalent response rates of 94.8% (CI 92.4–96.8%) and 92.1% (CI 88.6–95.0%), respectively (Ramai et al., 2020). Based on these findings, cFMT represents a less cumbersome, more practical, and more flexible approach for patients to restore gut microbiota diversity with the advantage that it can be administered in the outpatient setting. More standardized, oral products, such as SER-109, composed of purified *Firmicutes* spores (McGovern et al., 2021), are also showing promising results in preventing recurrence of *C. difficile* infection (Feuerstadt et al., 2022).

**Table 1:** Summary of published cFMT clinical trials in rCDI.

Study design	Outcome	Study type; country (reference)
Patients: 20 patients with rCDI. Primary outcome(s): safety, clinical resolution of diarrhea at 8 weeks. Secondary outcome(s): improvement in subjective wellbeing, number of daily bowel movements.	Primary results: no serious adverse events were reported. 90% (18/20) had clinical resolution of diarrhea without recurrence at eight weeks. 14/20 had resolution after one treatment, 4/6 had resolution after two. Secondary results: self-ranked health scores improved significantly. Daily number of bowel movements decreased from median of five to one per day at 8 weeks. Microbiota engraftment: not assessed. Additional findings: compared to NGT/colonoscopy delivery route, time to resolution was longer (2 days vs. four days) with cFMT.	Open-label, single-group, preliminary feasibility study; USA (Youngster et al., 2014a)
Patients: 116 patients with rCDI randomized to capsule (n = 57) or colonoscopy FMT (n = 59). Primary outcome(s): proportion of patients without rCDI at 12 weeks after FMT. Secondary outcome(s): adverse events, changes in quality of life, perception, and satisfaction with intervention.	Primary results: 96.2% prevention of rCDI after single treatment in both capsule (51/53) and colonoscopy (50/52) groups, meeting criterion for non-inferiority of cFMT. Secondary results: rate of minor adverse events was 5.4% in the capsule group vs. 12.5% in the colonoscopy group. There were no significant changes in quality of life between groups. Significantly more patients in the capsule group perceived the treatment as “not unpleasant” (66% vs. 44%). Microbiota engraftment: pre-FMT rCDI samples had a lower Shannon diversity index compared to donors. The Shannon diversity index increased to levels similar to donors in both capsule and colonoscopy groups post-FMT. PCoA of patients with rCDI clustered apart from donors prior to FMT and moved toward donor profiles post-FMT. PCoA clustering and alpha diversity were sustained at 12 weeks.	Non-inferiority, unblinded, randomized trial; Canada (Kao et al., 2017)
Patients: 65 patients with rCDI randomized to encapsulated, lyophilized FMT (n = 31) or frozen FMT by enema (n = 34). Primary outcome(s): Safety in 3 months post-FMT. Secondary outcome(s): prevention of rCDI during 60 days post-FMT.	Primary results: no differences in rate of adverse events between groups were observed. Secondary results: rCDI was prevented in 84% of patients randomized to capsules (26/31) and 88% who received FMT by enema (30/34), p = 0.76. Microbiota engraftment: the inverse Simpson index showed low diversity in pre-FMT samples, which increased post-FMT in both groups. Microbiota in patients receiving enema more rapidly resembled the donor composition in PCoA at as early as 2 days post-FMT, with both groups resembling donor composition by ninety days. Capsules were less effective in engrafting *Bacteroidia* and *Verrucomicrobia*.	Randomized, single-center clinical trial; USA (Jiang et al., 2018)
Patients: 537 patients with rCDI were included from 8 RTCs; fresh FMT (n = 273) vs. control (n = 264). The control group included patients who received antibiotic therapy, placebo, frozen, or capsule FMT. Primary outcome(s): clinical remission of diarrhea in without recurrence after 8 to 17 weeks. Secondary outcome(s): adverse events.	Primary results: recurrence rate of diarrhea in the fresh FMT group was 11% (30/273) vs. 24.6% (65/264) in the control group (p < 0.05). There was no difference in recurrence for patients treated with antibiotics or frozen FMT vs. fresh FMT by enema or those treated with cFMT and frozen FMT by colonoscopy vs. fresh FMT by colonoscopy. Secondary results: adverse events were mild to moderate and self-limited. There were no severe adverse events associated with FMT. Microbiota engraftment: not assessed.	Meta-analysis (Hui et al., 2019)

While cFMT has gained attention for its use in treating rCDI, it is also now being applied to a wide range of microbially associated diseases that may benefit from repopulation of the gut with a healthy microbiota (Sadowsky and Khoruts, 2016). cFMT is actively being studied in a wide range of conditions including inflammatory bowel disease (IBD), obesity/metabolic syndrome, and neurologic disorders, among others. In this review, we will discuss the development of the FMT capsule product, its clinical efficacy, and its pharmacokinetics in comparison to eFMT, as well as future directions for this restorative therapy.

## Development of the Capsule Product

Despite medical literature dating back to the fourth century, FMT research is still a developing procedure, specifically in regard to donor selection (Stripling and Rodriguez, 2018). When FMT first emerged in contemporary medicine, it was thought that individuals closely related to the patient would provide the optimal, fresh donor fecal microbiota for restorative repopulation (Bibbò et al., 2020). Patients were often tasked with finding a suitable donor; however, this presented a logistical difficulty and added to patient burden in addition to their illness. Moreover, no benefit was observed using fresh vs. frozen fecal preparations (Lee et al., 2016). In a practical effort to make donor fecal material readily available, the use of prescreened “universal” donors whose stool could be banked became a common practice internationally (Hamilton et al., 2012; Cammarota et al., 2019). However, recent concerns related to extended-spectrum beta-lactamase (ESBL) *Escherichia coli* and other multidrug-resistant species remain a concern when using banked stool and have resulted in patient death following FMT (DeFilipp et al., 2019). Therefore, it is critical for patient safety that donors and biobanked specimens are rigorously screened and checked regularly to ensure product safety.

Throughout its modern usage, though, the selection of the optimal donor in addition to screening protocols for safety has actively evolved and there is still a lack of clear resolution regarding the features of the optimal donor (Woodworth et al., 2017; Bibbò et al., 2020). Several small-scale studies have proposed the use of FMT super-donors, as FMT success has shown to be dependent on the composition and microbial diversity of the stool donor (Wilson et al., 2021). While this hypothesis is tempting, the absence of large, randomized clinical trials of FMT for the treatment of rCDI or other conditions suggests that the existence of FMT super-donors is yet to be supported by concrete empirical evidence. Nevertheless, an international consensus regarding the use of banked frozen donor material and consistently emerging recommendations may help standardize this practice (Cammarota et al., 2019).

The encapsulation of donor material for oral delivery represented the next practical step in improving both the aesthetics of FMT and its safety and availability. Similar to endoscopic and nasoduodenal approaches to administration, the first encapsulated FMT products were made from frozen donor preparations (Youngster et al., 2014a). These preparations were amended with 10% glycerol for ultralow-temperature storage, but uniformity of preparations and storage remained practical barriers to high-throughput usage. To address these concerns, our group pioneered the development of a freeze-dried preparation that would preserve the viability and diversity of the microbiota while at the same time reducing cumbersome mechanical barriers to capsule production (Staley et al., 2017a). These capsules were manufactured under Good Manufacturing Practice (GMP) standards, and use of the lyophilized powder did not significantly reduce the membrane integrity of the microbiota relative to fresh stool (Staley et al., 2017a). Due to a lack of standardized methods, emerging encapsulated FMT products vary somewhat in their formulation and to a greater degree in their mechanical properties, dosage, and delivery regimen, with the majority of capsules still made using frozen donor material (**Table 2**).

**Table 2:** Summary of published cFMT preparation methods.

Disease	Preparation of inocula	Capsule materials	Dose and # of patients	Storage	Reference
rCDI	Single-donor fecal slurry was concentrated by centrifugation and resuspended (1:10) in saline with 10% glycerol added as a bacterial cryoprotectant.	Encapsulated using commercially available acid-resistant hypromellose capsules (DRcaps, Capsugel–acid resistant). Inocula added to size 0 capsules (650 µl) were closed and then secondarily sealed in size 00 capsules.	15 capsules administered on two consecutive days.	Capsules were frozen and stored at −80°C.	(Youngster et al., 2014a)
rCDI	Single-donor sample mixed in 200 cc of 0.9% normal saline and filtered using a stomacher bag to produce 180 cc of fecal slurry. The slurry was mixed with 20 cc of 100% glycerol (10% final conc.).	Encapsulated using No. 1 gelatin capsules (1889-02; Medisca) then secondarily sealed with No. 0 (2009-02; Medisca) and No. 00 (1109-02; Medisca) capsules. Gelatin capsules used were not acid resistant.	Single dose of 40 capsules (360 ml of fecal microbiota total).	Capsules were flash frozen at −55°C on dry ice and stored at −70°C.	(Kao et al., 2017)
rCDI	100 grams of stool/donor donation were processed within 4 h of passage by mixing a 1:5 dilution in 500 ml of sterile 0.85% NaCl containing glycerol followed by filtering twice through double-layered woven gauze prior to lyophilization.	Encapsulated using 00-size acid-resistant capsules.	2 doses of 100 g of fecal microbiota each. 24 hours apart.	Capsules were stored at 4°C.	(Jiang et al., 2018)
IBS	12 g of fresh donor stool was frozen with 30% glycerol prior to encapsulation.	Double encapsulated using Capsugel DRcaps size 0 and 00.	25 capsules every morning for 12 days. Each daily dose contained 12 g frozen fecal material.	Capsules were frozen at −20°C and stored at 5°C.	(Halkjær et al., 2018)
SIBO	250 ml of sterile normal saline was added to 100–150 g of fecal matter for homogenization. After the slurry was filtered, trehalose, a cryopreservant, was added for lyophilization. The final bacterial concentration was 60 mg/ml.	Encapsulated using enteric-soluble capsules of 0.9 g/grain.	16 capsules once a week for 4 weeks.	Capsules were frozen and stored at -80°C.	(Xu et al., 2021)
Obese adults with mild to moderate insulin resistance	Fecal samples were suspended in saline and sieved. The slurry was then resuspended in saline at one-tenth the volume of the initial sample with 10% glycerol for freezing.	Encapsulated using size 0 capsules (650 µl), which were closed and then secondarily sealed in size 00 capsules.	15 capsules (for two consecutive days, then 15 capsules once a week for the following 5 weeks. Each capsule contained approximately 1.6 g; frozen fecal material.	Capsules were frozen and stored at -80°C.	(Yu et al., 2020)
Obese adults without diabetes, metabolic syndrome, or steatohepatitis	FMT preparation was performed using OpenBiome’s microbiota services. Donor stool was frozen with glycerol and glycerol before being encapsulated.	Double encapsulated in size 00 capsules with a gelatin interior capsule and an acid-resistant exterior capsule.	30 capsules were given as induction dose with maintenance dose of 12 capsules at weeks 4 and 8.	Capsules were stored at -20°C.	(Allegretti et al., 2020)
Obese and/or dyslipidemic adults	Fecal samples were suspended in saline and sieved. The slurry was then resuspended in saline at one-tenth the volume of the initial sample and frozen with 10% glycerol.	Encapsulated using size 0 capsules (650 µl), which were closed and then secondarily sealed in size 00 capsules.	10 capsules containing 1 g fecal microbiota material each were administered on ten occasions over a 6-month period (100 capsules total).	Capsules were frozen and stored at -80°C.	(Rinott et al., 2021)
Obese adolescents	Each capsule contained 0.25 g of fresh fecal matter pooled from four donors. The donor stool was then mixed with 0.5 ml of a cryoprotective saline solution (0.9% NaCl, 15% glycerol) and frozen.	Double encapsulated acid-resistant DRcaps™.	28 capsules over two consecutive days. Each capsule contained 0.25 g of microbiota.	Capsules were frozen and stored at -80°C.	(Wilson et al., 2021)
HIV	FMT preparation was performed using OpenBiome’s microbiota services. Donor stool was frozen with glycerol and glycerol before encapsulated.	Double encapsulated in size 00 capsules with a gelatin interior capsule and an acid-resistant exterior capsule.	10 capsules were given in the first dose followed by 5 capsules weekly for seven weeks. (45 capsules containing 30 g of stool total was given over eight weeks.).	Capsules were stored at -20°C.	(Serrano-Villar et al., 2021)

### Donor Material Preparation

Homogenization of the donor material is a ubiquitous first step to capsule preparation and is typically carried out in normal saline with dilution factors ranging from 5× to 10× (**Table 2**). Among frozen preparations, the donor slurry is typically amended with 10% glycerol. However, the use of freeze-dried preparations necessitated the use of different lyoprotectants to reach a practical viscosity for encapsulation (Staley et al., 2017a). Various lyoprotectants including sucrose, trehalose, mannitol, and skim milk, alone or in combination, at concentrations ranging from 2.5% to 10% were tested to identify which products provided an optimal consistency while maintaining bacterial viability. Milk was not suitable for pharmaceutical preparations due to potential allergic reactions and batch variability. Both trehalose and mannitol were found to produce preparations that were easily ground into powder and encapsulated. Trehalose was found superior in preserving bacterial viability, with membrane integrity tests comparable to frozen products (Staley et al., 2017a), and it appears to work consistently among other groups pursuing lyophilized encapsulated microbiota (Xu et al., 2021). While the use of trehalose has been suggested to enhance the virulence of some strains of *C. difficile* (Collins et al., 2018), there are several studies that show trehalose supplementation does not interfere with the clinical efficacy of cFMT (Saund et al., 2020; Buckley et al., 2021).

### Capsule Construction

The size and types of capsules used have also varied among different groups based on availability and the intended location of release (**Table 2**). However, the majority of studies have used size 00 capsules or smaller as a manageable size for patient use. When choosing the capsule material itself, the choice is dependent on the need for a specific location of release and engraftment. In order for the capsule to maintain its integrity as well as the bacterial viability, the use of acid-resistant encapsulation is crucial in its ability to provide protective properties against the harsh nature of the oral route of administration (**Table 2**). While most of the studies utilized an acid-resistant capsule and/or double encapsulation, this choice has varied considerably. In a study done to test the capsules’ ability to withstand the acidic environment present during the capsule transit, it was found that acid-resistant capsules were able to maintain membrane stability at a pH of 3 or less at 37°C before they began to leak (Varga et al., 2021).

Similar to the different choices of capsule material and size, the storage temperature of the encapsulated product varies among the studies (**Table 2**). The majority of the studies reported storage of their capsule products at -80°C for long-term storage, and -20°C along with 4°C for short-term storage. While it was known that frozen FMT material was required to be stored at ultralow freezing temperatures, the encapsulated product thermal stability has allowed some flexibility in storage. Lyophilized, encapsulated microbiota was tested in storage for 96 h at 20°C, 4°C, -20°C, and -80°C, with no significant differences in the microbiota viability as determined by membrane integrity (Staley et al., 2017a). This suggests that, at least among lyophilized preparations, short-term storage in patients’ homes may allow greater flexibility in FMT administration.

### Capsule Administration

Capsule administration provides a more flexible, less invasive, and more palatable option for FMT delivery without a reduction in clinical efficacy (Kao et al., 2017; Ramai et al., 2020). Colonoscopic administration benefits from the ability to visualize the colon and directly deliver the microbiota to the targeted area, and the ability to deliver larger quantities of microbiota; however, there is a risk due to the use of anesthesia as well as that of bowel perforation (Ramai et al., 2019). In comparison, upper gastrointestinal administration uses less stool but also has a greater risk of adverse events including aspiration, hemorrhage, and perforation (Wang et al., 2016; Ramai et al., 2020). Thus, in addition to increasing the flexibility of administration while reducing the unpleasantness associated with the procedure, cFMT may also represent a safer route of administration.

Capsule dosage and administration regimens have varied widely among studies (**Table 2**). The average total dose present in the studies ranged from 100 to 400 mg; however, the timing of administration and quantity of the capsules varied considerably based on study timelines and objectives. In our experience, doses ranging from 2.1 × 10^11^ to 2.5 × 10^12^ bacteria did not significantly affect clinical efficacy or the extent of microbiota engraftment (Staley et al., 2017a), nor did a prior bowel cleansing using polyethylene glycol (tested in four patients). However, several groups have also taken into account other medications that may interfere with microbiota engraftment, primarily proton pump inhibitors (PPIs) that may impair capsule opening (Kao et al., 2017). PPIs are commonly administered to lessen the symptoms of acid reflux and severe heartburn (Freston, 2004). Fortunately, multiple studies found no difference in clinical efficacy or engraftment among patients taking PPIs vs. patients not taking them (Youngster et al., 2014a; Staley et al., 2017a; Hong et al., 2020). The current weight of evidence suggests that, despite a number of potential confounding elements, oral administration of FMT is a relatively flexible and durable approach for microbiota restoration.

## Clinical Applications of cFMT

The gut microbiota has been linked to various other diseases, including inflammatory bowel diseases (IBD), neurologic disorders, and obesity, and there has been much enthusiasm around using FMT to target potential dysbiosis that may contribute to these conditions. While the clinical efficacy, non-inferior outcomes (>90% cure rate), and advantageous delivery method of cFMT in treating rCDI have been well established, the use of FMT in treating non-rCDI diseases is still a burgeoning area of interest. A PubMed search of clinical trials investigating cFMT revealed seven published studies after excluding those focused on rCDI (**Table 3**). These were all small, pilot studies and included irritable bowel syndrome (IBS), small intestinal bacterial overgrowth (SIBO), obesity, and HIV. A search of all currently registered cFMT clinical trials on Clinicaltrials.gov yielded over 100 results where cFMT is being evaluated in the treatment of a wide range of pathologies including rCDI, obesity and metabolic syndrome, IBD, IBS, cancers, HIV, the gut–brain–microbiota axis (in conditions such as depression and autism spectrum disorder), atopy and allergies, non-alcoholic fatty liver disease, hypertension, and graft-vs.-host disease, among others (**Table 4**). The efficacy of FMT in treating these diseases is less straightforward than in cases of rCDI, owing, in part, to increased complexity of diseases including multifactorial etiologies and lack of clear, infectious targets. Further studies of cFMT in these contexts may help expound on the pathologies of these diseases while also improving patient outcomes.

**Table 3:** Summary of published cFMT clinical trials in non-rCDI diseases.

Disease	Study design	Outcome	Study type; country (reference)
IBS	Patients: 52 patients with moderate-to-severe IBS randomized to cFMT (n = 26) or placebo capsule (n = 26). Primary outcome(s): change in disease severity, measured by IBS-SSS and IBS-specific quality of life, measured by IBS-QoL at 3 months. Secondary outcome(s): side effects.	Primary results: significant improvement in IBS-SSS and IBS-QoL was demonstrated with placebo. Secondary results: majority of patients in both groups experienced side effects. Patients in the cFMT group had significantly more diarrhea. Microbiota engraftment: stool was collected at 0, 1, 3, and 6 months for analysis. Alpha diversity at baseline was lower in IBS patients vs. fecal donors. Post-FMT IBS patients had increased alpha-diversity to the level of donors, while placebo remained similar to baseline. Alpha diversity did not correlate with IBS-SSS. PCoA showed post-FMT recipients grouped with the donor microbiota while placebo recipients did not. 11 donor OTUs were established in FMT recipients.	Double-blinded RCT; Denmark (Halkjær et al., 2018)
Obesity	Patients: 24 adults with obesity and mild to moderate insulin resistance were randomized to lean donor cFMT (n = 12) vs. placebo capsule (n = 12). Primary outcome(s): change in insulin sensitivity at 6 weeks. Secondary outcome(s): HbA1C, body weight, body composition, and resting energy expenditure was assessed at 6 and 12 weeks.	Primary results: non-significant improvement in insulin sensitivity in the cFMT group compared to the placebo group (9% increased in insulin-stimulated glucose uptake). Secondary results: no differences in fat mass, body weight, resting energy expenditure, or fasting lipids. Statistically significant greater, but clinically minor, reduction in HbA1C (-0.1 mean difference) in the cFMT group. Microbiota engraftment: alpha diversity in one donor was high compared to participants’ baseline, while the other three donors’ alpha diversity well within the interquartile range. Microbiota of FMT recipients were more similar in composition to donor microbiota than to their own baseline sample. FMT recipients exhibited engraftment of donor-specific sequence variants, although this was variable between those that received donor. FMT recipients did not display increased alpha diversity post FMT; however, those with low baseline alpha diversity had greater improvements in metabolic outcomes in the FMT group vs. placebo.	Double-blind RCT; USA (Yu et al., 2020)
Obesity	Patients: 22 obese patients without diabetes, metabolic syndrome, or steatohepatitis were randomized to cFMT (n = 11) or placebo (n = 11). Primary outcome(s): safety through week 26. Secondary outcome(s): changes in gut microbiome profile, bile acid profile, SCFA, change in obesity markers including GLP1, and leptin.	Primary results: no serious adverse events occurred. Secondary results: reduction in stool taurocholic acid was seen in the cFMT group. There was no change in SCFA in cFMT compared to placebo. GLP1 had an overall decrease in both groups and leptin was increased more in the placebo group. Microbiota engraftment: cFMT led to a non-significant increase in alpha diversity and significant change in beta diversity with shift to the lean donor profile. 200 OTUs were identified as engrafting from the donor; 9 were in the bile-hydrolyzing and butyrate-producing *Faecalibacterium* genus, which was depleted in obese patients compared to lean donor samples.	Double-blinded RCT; USA (Allegretti et al., 2020)
Obesity	Patients: 90 obese and/or dyslipidemic adults were randomly assigned to healthy diet (n = 16), Mediterranean diet (n = 35), or green-Mediterranean diet (n = 39) and who met inclusion criteria with >3.5% weight loss at 6 months were then randomly assigned to autologous cFMT vs. placebo. Primary outcome(s): weight regain at 14 months. Secondary outcome(s): GI symptoms, waist circumference, glycemic status, and changes in gut microbiota.	Primary results: no significant weight regain among all cFMT groups vs. placebo (30.4% *vs.* 40.6%). Significant decreased weight regain in the cFMT green-Mediterranean group vs. placebo (17.7% vs. 50%). Secondary results: the green-Mediterranean cFMT group had better glycemic control and decreased waist circumference gain vs. placebo. Microbiota engraftment: green-Mediterranean diet was the only group to show change in microbiota composition by PCoA at 6 months, and this was maintained only in the cFMT group at 14 months.	RCT; Israel (Rinott et al., 2021)
Obesity	Patients: 87 obese adolescents were randomized to receive cFMT or placebo. Primary outcome(s): bacterial strain engraftment in the setting of multiple donors over the 26-week period. Secondary outcome(s): N/A.	Primary results: see below. Secondary results: N/A. Microbiota engraftment: identified “super engrafter” donors that were highly effective at engrafting in the recipient gut using shotgun sequencing. These donors were characterized by high microbial diversity and high *Prevotella* to *Bacteroides* ratio. Despite the standard FMT dose, recipients had wide variability in engraftment of donor strains.	Double-blinded RCT; New Zealand (Wilson et al., 2021)
SIBO	Patients: 55 patients with moderate to severe SIBO were randomized to cFMT (n = 28) or placebo (n = 27). Primary outcome(s): improvement in GI symptoms rating scale (GSRS) and lactulose hydrogen breath test at 6 months. Secondary outcome(s): fecal microbiota diversity.	Primary result: significant improvement in GSRS after treatment with cFMT. Exhaled hydrogen was significantly decreased in the cFMT group compared to baseline. Secondary results: see below. Microbiota engraftment: donors had higher alpha diversity than patients with SIBO at baseline. The post-FMT group’s alpha diversity was more similar to that of donors. PCoA showed that cFMT recipients’ microbiota was also more similar to donors. *Bacteroides* abundance significantly increased in recipients of cFMT.	RCT; China (Xu et al., 2021)
HIV	Patients: 30 HIV-infected patients on ART were randomized to cFMT or placebo. Primary outcome(s): safety, adverse events. Secondary outcome(s): CD4 counts, CD4/CD8 ratio, inflammatory markers, enterocyte barrier function.	Primary results: no serious adverse events reported. Secondary results: no difference in CD4 counts, CD4/CD8 ratio, and inflammatory markers between groups. There was a significant decrease in intestinal fatty acid-binding protein (marker of intestinal damage) in the cFMT group. Microbiota engraftment: FMT attenuated HIV-associated dysbiosis. cFMT induced increased alpha diversity and transient engraftment of donor microbiota. *Lachnospiraceae* and *Ruminococcaceae* families more robustly engrafted over time, taxa depleted in HIV.	Pilot double-blinded RCT; Spain (Serrano-Villar et al., 2021)

**Table 4:** Compilation of registered cFMT trials by disease category.

Disease category	Number of registered trials
*Infectious*	*38*
rCDI	22
Carbapenem-resistant Enterobacteriaceae	8
HIV	5
Multidrug-resistant organism	2
COVID-19	1
*IBD/IBS*	*22*
Ulcerative colitis (UC)	12
IBS	4
Crohn disease	3
IBD + IBS	2
UC + Crohn disease	1
*Neurological/psychological disorders*	*10*
Depression	3
Autism spectrum disorder	2
Parkinson disease	2
Alzheimer disease	1
Schizophrenia	1
Multiple sclerosis	1
*Graft-vs.-host disease*	*7*
*Obesity/metabolic syndrome*	*6*
*Cancer*	*6*
Melanoma	2
Lung	1
GI	1
Colon + small intestine	1
Renal cell carcinoma	1
*Hematologic*	*4*
Bone marrow transplant	1
Hematopoietic stem cell transplant (HSCT)	1
Acute myeloid leukemia +HSCT	1
Immune thrombocytopenia	1
*Cirrhosis +/- hepatic encephalopathy*	*3*
*Peanut allergy*	*2*
*NAFLD*	*1*
*HTN*	*1*
*CKD*	*1*
*DM1*	*1*
*Chronic inflammatory disease*	*1*

### Inflammatory Bowel Disease

Alterations in host microbiota are thought to contribute to the multifactorial pathogenesis of IBD. Studies have shown that patients with ulcerative colitis (UC) and Crohn’s disease (CD) have reduced microbial diversity and decreased abundances of predominant phyla, specifically Firmicutes and Bacteroidetes (Vaughn et al., 2016; Levy and Allegretti, 2019). It is hypothesized that dysbiosis contributes to intestinal inflammation resulting in aberrant host immune responses and that normalizing the microbiota of IBD patients using FMT may improve symptoms and induce remission. Overall, the clinical efficacy of FMT in IBD patients has demonstrated equivocal outcomes (**Table 5**).

**Table 5:** FMT in IBD patients.

Disease	Study design	Outcome	Study type; country (reference)
UC and CD	Patients: 11 patients with IBD (11 UC, 3 CD). Formulation: 60 g fresh fecal material mixed with 350 ml saline *via* colonoscopy (UC) or NJT (CD). Dose: patients received 3 days of oral vancomycin and polyethylene glycol the day prior to the procedure. Primary outcome(s): quality of life based on IBDQ, improvement in CDAI score, and CRP (CD) or Mayo score (UC) at 4 weeks. Secondary outcome(s): patient attitude toward FMT	Primary results: all patients had remission of symptoms (bloody stool, fecal urgency, diarrhea) after 4 weeks. There were significant improvements in IBDQ scores, > in UC than CD (135 to 177 vs. 107 to 149). Mayo score decreased significantly (5.8 to 1.5, p < 0.1), CDAI decreased, but was not significant (345 to 135 (p = 0.082). Secondary results: patients did not wish to get repeated FMT and hoped for a pill formulation. Microbiota engraftment: not assessed.	Prospective, open-label uncontrolled trial; China (Wei et al., 2015)
UC and CD	Patients: 14 patients with refractory IBD (8 UC, 6 CD). Formulation: fresh stool was mixed with 400 ml saline stored at 4 C for 48 h prior to FMT. FMT was administered *via* NJT for the first 9 patients, then rectal tube for the last 5 immediately after colonoscopy. Dose: patients received polyethylene glycol bowel prep prior to colonoscopy for calculation of endoscopic score. 200 g of donor stool was used per patient. Primary outcome(s): improvement in CD Endoscopic Index of Severity (CDEIS), Simplified Endoscopic Activity Score (SES-CD), or Mayo endoscopic score at 8 weeks post-FMT. Secondary outcome(s): CD Activity Index (CDAI) or Mayo score, CRP.	Primary results: no significant improvement in CD patients. 2/8 UC patients had endoscopic remission at 8 weeks and 2 years, 1/8 had temporary remission at 6 weeks. Secondary results: there was an increase in CRP post-FMT in the UC group. No significant change was demonstrated in Mayo score or SES-CD. Microbiota engraftment: donor stool in the responders were found to have significantly more richness. There was no difference in transfer of donor phylum in the responders (74%) and non-responders (63%). *Roseburia* and *Oscillibacter* were transferred in the two responders only/.	Prospective, open-labeled uncontrolled trial; Belgium (Vermeire et al., 2016)
UC, CD, and IC	Patients: 21 pediatric patients with medically refractory IBD (UC, n = 12, CD, n = 7, or indeterminate colitis (IC) n = 2). Formulation: 150 g fresh stool per donor mixed with 250–300 ml saline. Dose: patients were pretreated with metronidazole or vancomycin and omeprazole for 5 days pre-FMT. Upper endoscopy was performed, and 20–30 ml of the fecal preparation was delivered to the duodenum or jejunum. Colonoscopy was then performed, and 200–250 ml fecal preparation was delivered to the terminal ileum and right colon. Primary outcome(s): safety. Secondary outcome(s): clinical response (decrease in PUCAI by 15 points or PCDAI by 12.5 points), remission (normalization of fecal markers, PUCAI/PCDAI score 0), and microbiota changes at 1 week, 1 month, and 6 months.	Primary results: no serious adverse events. Secondary results: 57% short-term response. 71% CD and 50% UC/IC were responders at 1 month. 43% CD and 21.4% UC/IC maintained response at 6 months. 2 CD patients had remission at 6 months. Microbiota engraftment: donor, pre-FMT, and post-FMT stool was analyzed. Alpha diversity was reduced in pre-FMT samples compared to donors. PCoA showed clustering of donors, while pre-FMT patients’ samples were more dispersed. Post-FMT samples had significantly increased alpha diversity at 1 month that decreased toward baseline at 6 months. Post-FMT microbial composition became more similar to donors at 1 month with decreased Jaccard distances but shifted to baseline at 6 months. There was no significant difference in alpha diversity or composition between responders and non-responders; however, responders had significantly increased alpha diversity and decreased phylogenetic distance from donors at 1 month, where this was not significant in non-responders. IBD patients had markedly elevated *Enterobacteriaceae* and paucity of *Lachnospiraceae.*	Prospective, open-label uncontrolled trial; USA (Goyal et al., 2018)
CD	Patients: 30 patients with refractory CD with HBI ≥7. Formulation: fresh or frozen FMT by upper endoscopy. Dose: single delivery of FMT by upper endoscopy to the midgut. Patients received mesalazine starting 1 week prior to FMT and continued it for 3 months post FMT. Primary outcome(s): clinical improvement and remission at 1 month. Secondary outcome(s): hemoglobin, serum lipid levels, CRP, ESR, immune cell composition changes.	Primary results: clinical improvement (87%) and remission (77%) peaked at 1 month post FMT. Secondary results: ESR and CRP decreased after FMT while serum IgM increased. Changes in T cell populations were observed after FMT. Hemoglobin and serums lipids increased after FMT. Microbiota engraftment: N/A.	Prospective, open-label uncontrolled trial; China (Cui et al., 2015)
CD	Patients: 9 pediatric patients with CD. Formulation: fresh FMT by NGT. Dose: patients were premedicated with rifaximin for 3 days, omeprazole the day prior, and MiraLAX for 2 days. 30 g donor stool was mixed with 100–200 ml saline. Stool preparation was given through and NGT. Primary outcome(s): safety and clinical efficacy (PCDAI score, CRP, fecal calprotectin) at 2, 6, and 12 weeks. Secondary outcome(s): none.	Primary results: all adverse events were mild except for one patients who had moderate abdominal pain after FMT. 7/9 patients were in clinical remission based on PCDAI score at 2 weeks. 5/9 patients were in remission at 6 and 12 weeks. CRP decreased in all but one patient. Fecal calprotectin did not improve. Secondary results: adverse events were mild to moderate and self-limited. There were no severe adverse events associated with FMT. Microbiota engraftment: two patients did not engraft, three had a gradual engraftment over 12 weeks, and two had engraftment by the second week. Unclear if clinical response correlated with engraftment; however, patients with least similar pre-FMT microbiota had best clinical response. Two patients had clinical deterioration and were found to have increased *E. coli* during flare.	Prospective, open-label uncontrolled trial; USA (Suskind et al., 2015)
CD	Patients: 19 adults with refractory CD. Formulation: 50 g donor stool was mixed with 250 ml saline and glycerol, then frozen at -80C. Dose: patients underwent bowel prep with magnesium citrate the day before FMT. Colonoscopy was performed, and thawed FMT material was delivered from the terminal ileum and distal. Primary outcome(s): clinical parameters including HBI decrease by >3 at 12 weeks. Secondary outcome(s): microbiota engraftment and immune cell, mucosal T-cell response.	Primary results: 11/19 patients (58%) had clinical response at week 4 and 6/11 (55%) had sustained response at week 12. Secondary results: significant increase in T-regulatory cells at 12 weeks post-FMT. Microbiota engraftment: species-level similarity between donor and recipient was significantly greater among responders than non-responders. Alpha diversity significantly increased post-FMT and increase was greater for responders.	Prospective, open-label uncontrolled trial; USA (Vaughn et al., 2016)
CD	Patients: 10 patients with CD. Formulation: single dose of frozen FMT *via* colonoscopy. Dose: unspecified lavage solutions were used to purge luminal content prior to FMT. 3/10 patients received rifaximin pretreatment. 250 ml FMT material was instilled at the terminal ileum. Primary outcome(s): clinical response (improvement in Harvey-Bradshaw index (HBI) score ≥ 3). Secondary outcome(s): clinical remission (HBI < 3), improvement in simple endoscopic score (SES), decreased ESR, CRP, fecal calprotectin, improvement in clinical symptoms at 1 month.	Primary results: 3/10 patients had HBI improvement ≥ 3. One patient had clinical remission. There were no significant changes in clinical parameters (pain, stool frequency, ESR, CRP, fecal calprotectin). No changes in SES between the responders and non-responders. Secondary results: rCDI was prevented in 84% of patients randomized to capsules (26/31) and 88% who received FMT by enema (30/34), p = 0.76. Microbiota engraftment: 16S rRNA sequencing from stool pre-FMT and 1 month post-FMT was compared to donors. Responders had lower alpha diversity at baseline, while there was no significant difference between non-responders and donors. Alpha diversity increased in 2/3 responders post-FMT. Post-FMT responders communities remained distinct from donors by pairwise comparison. Pre-FMT, 46 OTUs differed significantly between responders and non-responders. Post-FMT, 78 OTUs differed between the groups. Additional findings: longer disease duration was associated with responders. Study was terminated early due to adverse event of two patients having CD flare within days of FMT.	Prospective, open-label uncontrolled trial; USA (Gutin et al., 2019)
CD	Patients: 143 patients with CD. Formulation: fresh fecal preparation of 50 g microbiota in 100 ml saline delivered to mid-gut *via* endoscopy, NJT, or transendoscopic tubing. Dose: step-up FMT strategy was used; Step 1: single FMT; Step 2: ≥ 2 FMTs; Step 3: FMT(s) followed by steroids, immunomodulators or enteral nutrition. Primary outcome(s): clinical outcomes including response, remission, surgery, death, switching therapy, hematochezia, abdominal pain, fever, diarrhea, enterocutaneous fistula, perianal fistula, and steroid-dependence at 1, 3, 6, 12, 24, and 36 months after FMT. Secondary outcome(s): clinical response at 1 month after FMT.	Primary results: 75.3% (131/174) patients had clinical response after 1 month. 9.2% (12/131) had sustained remission after single FMT. 75.6% (109/131) underwent multiple FMTs; 58.7% had clinical response, 21.1% had sustained remission. 10.7% (14/131) switched therapy. 43.7% and 20.1% had clinical response and sustained remission, respectively, at final follow-up. Improvement in therapeutic targets at 1 month: abdominal pain 72.7%, diarrhea 61.6%, hematochezia 76%, fever 70.6%, steroid-free 50%, enterocutaneous fistula 80%, perianal fistula 33%. Secondary results: 75.3% (131/174) patients had clinical response after 1 month. Microbiota engraftment: not assessed. Additional findings: disease course of > 5 years was associated with non-responders.	Prospective, open-label uncontrolled trial; China (Xiang et al., 2020)
CD	Patients: 17 patients with CD were randomized to FMT (n = 8) or sham (n = 9). Formulation: fresh donor stool suspended in saline. Dose: patients receive 4 l polyethylene glycol prior to FMT. 50–100 g of stool from a single donor mixed with saline was delivered by colonoscopy. Primary outcome(s): colonization of donor microbiota at 6 weeks determined by Sorensen’s index > 0.6. Secondary outcome(s): feasibility of FMT, clinical flare rate at 24 weeks, steroid-free remission.	Primary results: the primary endpoint was not achieve in any recipient. Secondary results: flare rate was lower in the FMT than sham group but not statistically significant (3/8 in FMT group vs. 6/9 in sham group). Clinical remission was at week 10 was 7/8 (87.5%) in the FMT group vs. 4/9 (44.4%) in the sham group. Endoscopic index severity decreased significantly after FMT but not after sham. Microbiota engraftment: a transient, significant increase in diversity was observed after FMT but not sham. Two patients after FMT demonstrated failure of engraftment by Sorensen index. The remaining FMT patients had higher Sorensen index and increased proportion of donor OTUs. Failure of engraftment was associated with early flare. Taxa associated with engraftment failure, flare, and remission were identified.	RCT; France (Sokol et al., 2020)
UC	Patients: 75 patients with active UC. Formulation: randomly assigned 50 ml FMT by enema (n = 38) or placebo water enema (n = 37). Dose: 50 g donor was mixed with 300 ml bottled water. FMT was instilled by enema immediately or stored at -20°C. Enema was given once a week for 6 weeks. Primary outcome(s): remission of UC defined by Mayo score ≤ 2 and endoscopic Mayo score of 0 at 7 weeks. Secondary outcome(s): IBD questionnaire and sigmoidoscopy with biopsies at week 7.	Primary results: 9/38 (24%) of patients who received FMT and 2/37 (5%) who received placebo were in remission at 7 weeks (p = 0.03). 8/9 remained in remission at1year. Significantly higher proportion of patients with UC for < 1 year achieved remission versus those with chronic UC. Secondary results: 7 patients in remission had no inflammation on biopsy at 7 weeks post FMT and 2 had patchy inflammation. Microbiota engraftment: 7/9 patients in remission received stool from a single donor. This donor was enriched in family Lachnospiraceae and *Ruminococcus.* Patients receiving FMT had greater diversity compared to baseline than those who received placebo. Post-FMT stool was significantly more similar to donor stool than baseline.	RCT; Canada (Moayyedi et al., 2015)
UC	Patients: 48 patients with UC were randomized to donor FMT (n = 23) *vs.* autologous FMT (n = 25). Formulation: 500 ml fresh fecal suspension was administered *via* NDT. Dose: patients received 2 l macrogol solution the evening and morning prior to FMR. FMT was administered in two doses 3 weeks apart. Primary outcome(s): clinical remission determined by improvement in colitis activity index score and Mayo endoscopic score at week 12. Secondary outcome(s): clinical response, safety, and microbiota composition.	Primary results: remission was achieved in 7/23 (30.4%) patients in the donor FMT group and 5/25 (20%) in the autologous FMT group (p = 0.51). Secondary results: 11/23 (47.8%) of donor FMT patients and 13/25 (52%) in the autologous FMT group had clinical response. Serious adverse events occurred in 4 patients not related to FMT. Microbiota engraftment: microbiota of responders post-FMT shifted toward donor composition at 12 weeks. Alpha diversity increased in responders. Remission was associated with increased *Clostridium* clusters IV and XIVa.	RCT; Netherlands (Rossen et al., 2015)
UC	Patients: 82 patients with active UC were randomized to FMT (n = 43) vs. placebo (n = 43). Formulation: 37.5 g fecal material from three to seven pooled donors with saline and glycerol frozen at -80°C administered *via* lower endoscopy and enema. Dose: Patients underwent pre-procedure bowel preparation. The first FMT dose was infused by colonoscope. Subsequent doses were administered by enema five times per week for 8 weeks. Primary outcome(s): steroid-free clinical and endoscopic remission at week 8. Secondary outcome(s): steroid-free clinical response, clinical remission, endoscopic response, endoscopic remission, quality of life, safety.	Primary results: 11/41 (27%) of FMT recipients and 3/40 (8%) of placebo recipients achieved the primary outcome (RR 3.6, *p* = 0.02). Secondary results: clinical remission was 44% vs. 20%, clinical response was 54% vs. 23%, and endoscopic response was 32% vs. 10% in FMT vs. placebo patients (all significantly higher in the FMT group). Endoscopic remission was 12% vs. 8% in FMT vs. placebo groups (not significant). The was no significant difference in quality of life between groups. 78% of FMT and 83% of placebo patients experienced adverse events. Six serious adverse events occurred; there was no difference in adverse events between groups. Microbiota engraftment: diversity was significantly higher in donors than recipients. Patient treated with FMT had a significant increase in diversity from baseline. Principle coordinate analysis showed shift of recipients to donor composition with decrease in *Bacteroides* and increase in *Prevotella.* Several taxa were associated with remission including *Parabacteroides*, *Clostridium IV*, *Ruminococcus*, and *Blautia.*	RCT; Australia (Paramsothy et al., 2017a)
UC	Patients: 73 patients with active UC were randomized to receive anaerobically prepared pooled donor FMT (n = 38) or autologous FMT (n = 35). Formulation: stool was pooled from three to four donors, mixed with saline and glycerol under anaerobic conditions, and frozen at -80C. Dose: Patients received 3 l polyethylene glycol the evening before and loperamide immediately prior to colonoscopy. Initial dose of 50 g of stool was given by colonoscopy followed by two enemas containing 25 g of stool was given during the next 7 days. Primary outcome(s): steroid-free clinical and endoscopic remission at 8 weeks. Secondary outcome(s): clinical response, clinical remission, endoscopic remission, patient perception, colonic lamina propria mononuclear cell analysis, adverse events, and microbiota changes.	Primary results: clinical and endoscopic remission was attained in 12/38 (32%) polled donor FMT patients and 3/35 (9%) autologous FMT patients (OR 5, *p* = 0.03). Secondary results: clinical response was 55% vs. 23%, clinical remission was 47% vs. 17% in donor FMT vs. autologous FMT patients (both significantly higher in the donor group). Endoscopic remission occurred in 11% of donor FMT vs. 0% of autologous FMT patients (p = 00.12). There were three serious adverse events in the donor FMT group and two in the autologous FMT group. There were no significant changes in lamina propria mononuclear cells after FMT. Microbiota engraftment: microbial diversity was lower in recipient than donor stool. Diversity significantly increased in the donor FMT group compared to the autologous FMT group. Increase in anaerobic bacteria was seen after donor FMT. Increased abundance of *Anaerofilum pentosovorans* and *Bacteroides coprophilus* was associated with clinical improvement.	RCT; Australia (Costello et al., 2019)
UC	Patients: 12 patients with active UC were randomized to eFMT + cFMT (n = 6) or placebo (n = 6). Formulation: initial colonoscopy infusion of followed by 12 weeks of frozen cFMT. Dose: patients were pretreated with ciprofloxacin and metronidazole for 7 days prior to FMT and underwent unspecified standard bowel preparation. 48 g stool was infused *via* colonoscope and subsequently received 0.5 g stool daily by frozen cFMT. Primary outcome(s): clinical and endoscopic response at 12 weeks. Secondary outcome(s): T-cell composition, microbiota changes, adverse events.	Primary results: 2/6 patients in the FMT group and 0/6 in the placebo group achieve remission. Secondary results: T-cell changes were observed in the FMT group; however, there was not enough power to assess statistical significance. Four adverse events occurred evenly distributed between the groups; two were serious adverse events. Microbiota engraftment: no increase in diversity was seen after FMT, but the recipients’ composition became more similar to donors.	RCT; USA (Crothers et al., 2021)

There are five randomized clinical trials (RCTs) that investigated FMT in UC (Moayyedi et al., 2015; Rossen et al., 2015; Paramsothy et al., 2017a; Costello et al., 2019; Crothers et al., 2021). One small pilot RCT has been conducted evaluating FMT in CD (Sokol et al., 2020), but most data come from open-label cohort trials. In all IBD studies, FMT was delivered by enema, colonoscopy, or NDT; only one used cFMT, but this was in conjunction with an initial dose delivered by colonoscopy (Crothers et al., 2021). A meta-analysis found remission rates of 36% (201/555) in UC, 50.5% (42/83) in CD, and 21.5% (5/23) in pouchitis (Paramsothy et al., 2017b). When only RCTs in UC were analyzed, a significant benefit from FMT was found with odds ratio (OR) of 2.89 (*p* = 0.006). In subanalyses, greater rates of remission were associated with delivery *via* lower endoscopy and greater number of eFMT infusions received. Although no studies have been published yet regarding the efficacy of cFMT in IBD, there are currently 18 registered trials investigating this subject (**Table 4**). Evidence thus far suggests that FMT may be an efficacious treatment for UC. It is more difficult to determine its effect in CD and pouchitis given the lack of RCTs. More data are needed about FMT in the IBD population, in general, before any conclusions can be drawn about its role in future clinical practice.

### Neurological and Psychiatric Disorders

The gut–brain–microbiome axis refers to the bidirectional communication of the central nervous system and the gut microbiota *via* metabolites, hormones, and immunomodulators (Martin et al., 2018). Perturbations in this circuit are thought to contribute to a host of diseases, such as obesity, IBS, anxiety, depression, Parkinson’s disease (PD), and autism spectrum disorders (ASD). Use of FMT to restore a more “normal” brain–gut axis and alleviate symptoms of neurologic and psychiatric disorders is under investigation.

Preliminary clinical studies in patients with ASD have found promising results related to improvement of GI symptoms after FMT. Adults and children with ASD often have GI symptoms, including constipation and/or diarrhea, abdominal pain, and indigestion concomitant with behavioral symptoms (social skill and communication deficits, irritability, hyperactivity, repetitive behaviors, etc.), and these have been found to correlate in severity (Kang et al., 2017). Children with ASD have altered gut microbiota compared to children without ASD, leading to the hypothesis that the dysbiotic gut microbiota, potentially due to increased antibiotic use in early childhood, leads to changes in GI function and alteration in metabolites produced by microbiota, impacting neurobiological pathways (Kang et al., 2017). In a non-randomized, open-label clinical trial performed on 18 children and adolescents with ASD who underwent a 10-week course of FMT delivered *via* rectal administration or an oral powder mixed with chocolate milk, GI symptoms were significantly reduced by 80%, and gradually over the course of the study period, ASD-related behaviors also significantly improved. These improvements were sustained over the follow-up period of 8 weeks (Kang et al., 2017). Engraftment, measured by UniFrac distance (Lozupone and Knight, 2005), increased community diversity, which was lower at baseline in ASD subjects compared to non-ASD controls, and increased abundances of *Bifidobacterium* and *Prevotella* were observed. In a 2-year follow-up study of this cohort of patients, GI symptoms remained significantly improved and ASD-related behaviors continued to improve after the end of the treatment period (Kang et al., 2019). Analysis of plasma and fecal metabolites demonstrated significant changes in plasma metabolites from baseline after FMT, including increased nicotinamide riboside and IMP and decreased caprylate and heptanoate, suggesting a potential biochemical cause for symptoms of ASD (Kang et al., 2020).

The contribution of the gut–brain–microbiome axis to psychiatric diseases is another area of active study, and the role of FMT in major depressive disorders and anxiety disorders is being explored. A recent review article identified eight clinical trials assessing the effect of FMT on depression and anxiety symptoms (Chinna Meyyappan et al., 2020). While three of these studies were case reports and six primarily examined other disorders (i.e., IBS) with depression or anxiety symptom relief as secondary outcomes, these trials demonstrated significant improvements in short-term depression and/or anxiety, but variable long-term results (Chinna Meyyappan et al., 2020). Further study is warranted to clarify the role of FMT in psychiatric illnesses given the high prevalence of these diseases, stigmatism and morbidity associated with them, and need for effective treatment options.

Parkinson’s disease is a neurodegenerative disorder characterized by motor (tremor, bradykinesia, rigidity, shuffling gait) and non-motor symptoms, in particular constipation, in up to 80% of cases. Dysbiosis leading to alterations of the gut–brain axis has been implicated in the development of PD symptoms, and recent preliminary clinical studies have demonstrated that FMT can ameliorate constipation and improve motor symptoms (Huang et al., 2019; Xue et al., 2020; Kuai et al., 2021).

While there are no published cFMT trials in the context of neurologic and psychiatric diseases, a number are ongoing (**Table 4**). An encapsulated formulation is pertinent to the needs of this specific population of patients for whom invasive medical therapies may be especially taxing due to behavioral or mobility issues, frailty, anxiety, and agoraphobia.

### Obesity/Metabolic Syndrome

The basis of obesity, thought to be due to a complex interplay of environmental and genetic factors, is still not fully understood. Obesity is increasingly common and has been linked to disease states associated with the metabolic syndrome including type II diabetes and non-alcoholic fatty liver disease. Abnormalities in the gut microbiota have been linked to obesity: lower baseline species alpha diversity is often observed in obese individuals as well has a higher ratio of *Firmicutes* to *Bacteroidetes* compared to lean individuals (Napolitano and Covasa, 2020). Obesity-associated microbiota may contribute to increased caloric absorption and energy production, chronic inflammation and immune responses leading to insulin resistance, and dysregulated fatty acid metabolism (Napolitano and Covasa, 2020). While FMT studies conducted in obese and lean mice have robustly demonstrated the transferability of the obese phenotype, definitive clinical results have not been borne out in human trials. In a 2012 study, FMT delivered by NDT from lean donors to obese males with impaired fasting glucose resulted in significantly improved peripheral insulin sensitivity compared to obese recipients of autologous FMT (Vrieze et al., 2012). However, there was no change in weight, body mass index (BMI), glycated hemoglobin, resting energy expenditure, or glucoregulatory hormones between recipients of lean donor FMT and controls. A subsequent follow-up study in obese males who received either lean donor or autologous FMT through NDT infusion revealed significant improvement in peripheral insulin sensitivity among lean-donor recipients and marginal improvements in glycated hemoglobin at 6 weeks post-FMT (Kootte et al., 2017). Again, there were no changes in weight, BMI, resting energy expenditure, or enteroendocrine hormones between the two groups. Obese individuals who received lean-donor FMT had a significant increase in the fecal short-chain fatty acid (SCFA) acetate, which is inversely correlated with insulin resistance. Additionally, lean-donor recipients demonstrated an increase in the acetate-producing species *Bifidobacterium pseudolongum* within the duodenum, pointing to the potential role of microbiota-mediated SCFA production on insulin sensitivity in patients with metabolic syndrome. An increase in plasma amino acid γ-aminobutyric acid, a metabolite that promotes insulin sensitivity in rodent models, was also noted among recipients of lean-donor FMT. Lastly, the authors of this study noted that lower baseline fecal microbiota diversity predicted clinical response to FMT treatment.

Four trials investigating obesity and metabolic parameters using cFMT have been published to date (**Table 3**). Two recent studies showed no significant improvements in BMI or metabolic parameters after intervention (Allegretti et al., 2020; Yu et al., 2020). One study, which examined maintenance of weight loss after autologous cFMT within patients who underwent different dietary interventions, showed mixed results with significantly decreased weight regain after autologous cFMT in only one of the dietary intervention groups (Rinott et al., 2021). The most recent study (Wilson et al., 2021) examined engraftment after cFMT in obese adolescents but did not interrogate clinical or biochemical metabolic parameters. Although there was no clinical improvement in these trials, there was evidence of microbiota engraftment in all four studies. It is likely that the lack of clinical improvement in obesity and metabolic syndrome after cFMT is not due to a failure of the capsule technology itself, but rather that the efficacy of FMT in general is less well-established in obesity, as demonstrated in the equivocal results of the human FMT trials (Vrieze et al., 2012; Kootte et al., 2017). While dysbiosis exacerbates the development of obesity through various established pathways (i.e., microbial energy harvest, SCFA production, inflammation), obesity is a multifactorial disease; more data are needed to determine who will most likely benefit from FMT and what aspects of obesity-associated metabolic syndrome can be improved by manipulation of the microbiota.

### Other Conditions

cFMT is under investigation in a multitude of other non-rCDI diseases, including IBS, drug-resistant organisms, hematologic disorders, graft-*versus*-host disease, malignancies, and allergy (**Table 3**). Only a few studies have been published at this point. One completed study investigating cFMT in IBS (Halkjær et al., 2018) showed no improvement in clinical parameters; however, it did demonstrate microbiota engraftment. Another published study investigated HIV patients (Serrano-Villar et al., 2021) and found that cFMT did not lead to improved CD4 counts or inflammatory markers but did resolve dysbiosis typically associated with HIV and led to a decrease in biomarkers of intestinal injury. Many of the cFMT studies in non-rCDI diseases were small-scale, pilot studies limited in their statistical power (**Table 3**). In the future, RCTs with larger cohorts are needed to better evaluate the efficacy of cFMT in these various disease states. Another challenge that needs to be simultaneously addressed is to optimize and standardize capsule formulation and dose for more rigorous inter-study evaluations.

## Pharmacokinetics of cFMT

The pharmacology of FMT, an active biological community, is an emerging discipline with distinct principles from that of conventional drugs. The pharmacology of FMT must be understood in a framework of microbial ecology that considers the complex and dynamic interactions of gut microbiota with host factors such as diet, medications, and lifestyle, and relationships with other microorganisms (Khoruts et al., 2021). The standard concepts of pharmacokinetics (absorption, distribution, metabolism, and excretion) are poorly applicable to FMT therapies given that the “drug” is a metabolically active, complex consortium. Thus, FMT pharmacokinetics could be thought of largely in terms of microbiota engraftment. As previously discussed, engraftment is affected by many steps along the way in cFMT product formulation, from donor selection to the choice of preservation method to encapsulation strategy. In this section, rather than focusing on traditional pharmacological principles [reviewed recently (Khoruts et al., 2021)], we will discuss the pharmacokinetics of FMT as the kinetics of microbiota engraftment.

The assessment of microbial engraftment is an often overlooked or oversimplified metric in studies of FMT. Early studies relied on detection of taxonomic units, often as broadly as phyla, in post-FMT patient samples that were also present in the donor sample (Hamilton et al., 2013; van Nood et al., 2013; Seekatz et al., 2014; Kelly et al., 2016). Engraftment was established as a return of alpha diversity (richness and evenness) and a taxonomic distribution of bacteria dominated by members of the Firmicutes and Bacteroidetes phyla. However, high-resolution taxonomic compositions (i.e., species and strains) were not considered, partially due to limitations of the 16S rRNA gene amplicon sequencing read length, as well as potential non-detects based on limits of detection. Methods were refined to assess correlations between donor and recipient fecal communities (Weingarden et al., 2015; Jalanka et al., 2016), which provided a statistical evaluation of engraftment while still suffering from similar technical limitations. More recently, our group and others have utilized Bayesian approaches to infer levels of engraftment and invasion by specific taxa following FMT or animal cohousing (Ridaura et al., 2013; Khanna et al., 2017; Staley et al., 2017b; Le Bastard et al., 2018; Sokol et al., 2020; Haifer et al., 2021). Packages like SourceTracker (Knights et al., 2011) that employ this approach have been shown to be able to differentiate individual donor samples (Staley et al., 2018), determine taxa associated with engraftment (Ridaura et al., 2013; Staley et al., 2017b), and may be applied to emerging metagenomics datasets for use with high resolution taxonomic data. Below, we will discuss the current state of encapsulated microbial engraftment kinetics assessed using robust computational methods for quantitative assessment, with a focus on human studies of rCDI and clinical response to therapy.

### Kinetics of Engraftment in cFMT vs. eFMT

One of the striking differences in microbial engraftment between cFMT and more traditional eFMT is a delay in engraftment following cFMT relative to eFMT, despite similar clinical efficacy (Staley et al., 2017b; Jiang et al., 2018). Administration of frozen donor fecal material *via* colonoscopy has been shown to result in donor-like normalization of the patient microbiota within 1 week following administration by either colonoscopy or NDT (Hamilton et al., 2013; van Nood et al., 2013; Weingarden et al., 2015; Jalanka et al., 2016), and complete engraftment may occur within the first 48 h following FMT (Weingarden et al., 2015; Jiang et al., 2018). In contrast, cFMT using lyophilized preparations of donor microbiota resulted in slower, punctuated engraftment, and engraftment levels similar to those seen with eFMT were not observed until 2–4 weeks following administration (Staley et al., 2017b; Jiang et al., 2018). This delay was reflected in slower expansion of the Bacteroidetes, which corresponded with a shift from predominantly primary to secondary fecal bile acids (Staley et al., 2017b), and may reflect a need for a greater concentration of bacteria using this method. To address the incongruence between clinical efficacy and engraftment kinetics following cFMT, we investigated whether early signatures of engraftment were predictive of clinical outcomes and found that relative abundances of members of predominant families *Bacteroidaceae*, *Ruminococcaceae*, and *Lachnospiraceae* were highly predictive of clinical response (Staley et al., 2018), suggesting that methods to improve engraftment and expansion of these groups may be a promising clinical target.

The reasons for the delay in engraftment between cFMT and eFMT have become an active area of research with primary foci on improving microbiota formulation and determining an optimal method for delivery. Notably, in a multicenter, unblinded, RCT, differences in microbiota engraftment, determined by qualitative taxonomic comparison, did not appear to be prominent when frozen microbiota were delivered by colonoscopy or capsule (Kao et al., 2017). These results suggest that a feature of the lyophilization process may impair the early resuscitation of certain members of the microbial community, e.g., members of phylum Bacteroidetes. However, a recent study by our group using identical preparations of lyophilized microbiota delivered colonoscopically or orally indicated that the route of administration was also a significant variable, with greater levels of donor similarity observed following the former delivery method within the first 2 weeks (Staley et al., 2021). Given discrepancies in capsule preparation and data analysis, definitive conclusions regarding the reasons underlying delayed engraftment of lyophilized microbiota following cFMT remain to be determined. Nevertheless, results of current studies suggest a definite possibility to improve formulation methods to overcome limitations caused by both lyophilization and oral delivery.

### Durability of Engraftment Is Comparable in cFMT

The reconstitution of the donor microbiota following colonoscopic FMT has been investigated in a small subset of studies and was reported as generally stable over the period of 1 year (Weingarden et al., 2015; Jalanka et al., 2016). While fluctuations in the microbiota composition were observed, these were similar to those observed in healthy donors (Weingarden et al., 2015). However, even in cases of eFMT, donor engraftment is not always immediate, with one patient showing increased relative abundances of *Firmicutes* for 7 months following treatment before microbiota composition began to resemble that of the donor up to 4.5 years post-FMT, despite durable therapeutic success (Broecker et al., 2016). Following cFMT using lyophilized microbiota, donor engraftment of bacteria was recently reported to remain high for 6 months following FMT, although no evidence of fungal engraftment was observed (Haifer et al., 2021). Similarly, work by our group indicated that cFMT responders who received lyophilized microbiota maintained similarity to their donor for up to 1 year and patients receiving different cFMTs from differing donor lots could be significantly differentiated (Staley et al., 2019). We also noted three predominant patterns in engraftment—among 18 patients who responded to cFMT, 61% showed high (>50% donor similarity) and sustained engraftment, 22% showed high engraftment during the first month that later declined, and 17% showed very slow engraftment, reaching a maximum of <40% similarity by 1 year. We noted that abundances of *Bacteroides* and *Parabacteroides* were correlated with engraftment rate but typically reflected engraftment of only 2–4 strains, determined by oligotype analysis (Staley et al., 2019). Long-term engraftment following cFMT using frozen microbiota remains to be investigated, but early evidence suggests that cFMT results in durable long-term engraftment, similar to eFMT, in the absence of antibiotic and other provocations in the majority of patients.

## Discussion

Encapsulated FMT is becoming a mainstream therapeutic option to treat rCDI, with applications to a variety of other conditions in which FMT has been recently explored or thought to be of benefit (Sadowsky and Khoruts, 2016). While there is still a paucity of data reflecting the use of cFMT in conditions other than rCDI, the ease of administration and potential for storage outside of ultra-low-temperature conditions make it a promising avenue to expand the therapeutic reach of FMT. Similar to existing concerns regarding the standardization of FMT materials (Cammarota et al., 2019), methods of capsule preparation remain highly variable among research groups, reflective predominantly of practical concerns. Systematic investigations of parameters such as the role of cyro-protectants, capsule coating, and mechanical features to accommodate gastric transit and control capsule opening are necessary to determine critical aspects of capsule formulation to improve clinical efficacy and standardize production. In addition to efficacy, understanding and optimizing microbiota engraftment and expansion of critical taxa will be necessary, especially in the majority of conditions in which antibiotic exposure is not a first-line treatment. Abiotic parameters such as metabolite (e.g., short-chain fatty acids or bile acids) concentrations may also contribute to the success and kinetics of bacterial engraftment (Staley et al., 2017b), in addition to physical parameters of the capsule, and represent further areas of study to improve encapsulate microbiota therapeutics.

Early experience with cFMT in rCDI has highlighted a paucity of data regarding the role of microbial ecology in the clinical success of FMT. In relation to standard pharmacology, the application of a metabolically active, complex consortium as a therapeutic raises similar concerns about off-target effects related to the interaction of the microbiota with other drugs, diet, and probiotics (Khoruts et al., 2021). As the use of FMT is expanded as a potential treatment in other conditions, evaluating these interactions will be increasingly important both to improve clinical efficacy and over deleterious off-target effects. Donor screening is now increasingly considered with a hypothesis that a specific microbiota consortium may prove more beneficial in correcting dysbiosis underlying a specific disease; e.g., bacterial sulfur metabolism related to IBD (Bryant et al., 2021). In addition, understanding the competitive dynamics associated with engraftment and invasion will be necessary to optimize formulation, dosing strategies, and adjunctive therapies to improve outcomes following FMT.

## Author Contributions

HH and SB drafted the manuscript. CS provided the critical commentary and feedback. All authors contributed to the article and approved the submitted version.

## Conflict of Interest

The authors declare that the research was conducted in the absence of any commercial or financial relationships that could be construed as a potential conflict of interest.

## Publisher’s Note

All claims expressed in this article are solely those of the authors and do not necessarily represent those of their affiliated organizations, or those of the publisher, the editors and the reviewers. Any product that may be evaluated in this article, or claim that may be made by its manufacturer, is not guaranteed or endorsed by the publisher.
